# Intersection of Race and Rurality With Health Care–Associated Infections and Subsequent Outcomes

**DOI:** 10.1001/jamanetworkopen.2024.53993

**Published:** 2025-02-03

**Authors:** Katelin B. Nickel, Hannah Kinzer, Anne M. Butler, Karen E. Joynt Maddox, Victoria J. Fraser, Jason P. Burnham, Jennie H. Kwon

**Affiliations:** 1 Department of Medicine, Division of Infectious Diseases, Washington University School of Medicine, St Louis, Missouri; 2 Brown School, Washington University in St Louis, St Louis, Missouri; 3 Department of Surgery, Division of Public Health Sciences, Washington University School of Medicine, St Louis, Missouri; 4 Department of Medicine, Division of Cardiology, Washington University School of Medicine, St Louis, Missouri; 5 Center for Advancing Health Services, Policy & Economics Research, Washington University School of Medicine, St Louis, Missouri

## Abstract

**Importance:**

Health care–associated infections (HAIs) are a major cause of morbidity and mortality, but little is known about whether structural factors impacting race and rurality are associated with HAI and subsequent outcomes.

**Objective:**

To evaluate the association of race and rurality, which are proxies for structural disadvantage, with HAI and subsequent outcomes.

**Design, Setting, and Participants:**

This cohort study was conducted at 3 US urban and suburban hospitals. Participants were adults aged 18 years or older admitted for 48 hours or longer from January 1, 2017, to August 31, 2020. Statistical analysis was performed from November 2022 to April 2024.

**Exposure:**

Patient race and rurality status were defined as the combination of race (Black or White) and residence (urban or rural per patient zip code).

**Main Outcomes and Measures:**

HAI was defined as a positive culture from a urine, blood, or respiratory specimen obtained 48 hours or longer after admission. To determine the association of race and rurality with HAIs, multivariable generalized estimating equations models were used to account for clustering of admissions by patient. Among patients with HAI admissions, similar models examined post-HAI intensive care unit admission and in-hospital death.

**Results:**

Among 214 955 patients admitted to the hospital (median [IQR] age, 63 [51-73] years; 108 679 female patients [50.6%]; 72 490 Black patients [33.7%]; 142 465 White patients [66.3%]), recognized HAIs occurred during 6699 (3.1%). Compared with White urban patients, Black urban patients had a decreased risk of HAI (adjusted relative risk [aRR], 0.81; 95% CI, 0.75-0.87), White rural patients had an increased risk of HAI (aRR, 1.12; 95% CI, 1.05-1.20), and Black rural patients (aRR, 1.08; 95% CI, 0.81-1.44) had a similar risk of HAI. Among patients with HAI admissions, Black rural patients had an increased risk of intensive care unit admission (aRR, 1.92; 95% CI, 1.16-3.17) and in-hospital death (aRR, 1.78; 95% CI, 1.26-2.50). White rural and Black urban patients had outcomes similar to those of White urban patients.

**Conclusions and Relevance:**

This cohort study of hospitalized adults identified inequities related to race and rurality in HAIs and adverse outcomes from HAIs. These findings suggest that factors such as structural racism and disinvestment in rural communities may be associated with individual HAI risk and post-HAI outcomes. Future work to further understand the reasons underpinning these disparities and methods to address structural factors through policy and process changes are critical to eliminate health inequities.

## Key Points

**Question:**

Are race and rurality, which are proxies for structural disadvantage, associated with health care–associated infections (HAIs) and subsequent outcomes?

**Findings:**

In this cohort study of 214 955 hospitalized adults, Black urban patients had a decreased risk of recognized HAI and White rural patients had an increased risk of recognized HAI compared with White urban patients. Among HAI admissions, Black rural patients had a markedly increased risk of intensive care unit admission and in-hospital death compared with White urban patients.

**Meaning:**

These findings suggest that factors such as structural racism and disinvestment in rural communities may be associated with individual HAI risk and post-HAI outcomes, and future work should focus on addressing structural factors through policy and process changes to eliminate health inequities.

## Teaser

This cohort study examines the association of race and rurality, which are social determinants of health, with health care–associated infections and subsequent outcomes.

## Introduction

Social determinants of health (SDOH) are nonbiological factors impacting health that are outside an individual’s control.^1,2^ Conceptually, SDOH include social and community context, economic stability, neighborhood and built environment, health care access and quality, and education access and quality.^1,2^ Systemic and structural factors that shape SDOH in ways that advantage certain populations and disadvantage others underlie health disparities and inequities.^1,3,4^

Black individuals in the US live in a context shaped by individual, structural, and systemic racism.^5^ Structural racism impacts all SDOH domains; for example, numerous laws and policies, from unequal housing and lending policies known as redlining to segregated education and health care facilities, have contributed to differential health care access and quality.^4,6,7,8,9,10,11^ These factors have collectively led to persistent and pervasive racial inequities in health outcomes. For instance, Black populations experience higher rates of cardiovascular disease, stroke, and certain cancers, as well as lower life expectancy.^3,12,13,14^

Systemic and structural factors have also led to health inequities in rural populations. People living in rural areas have less access to health care, longer distances to specialty and emergency care, reduced preventive health care utilization (eg, cancer screening and vaccination), and a higher burden of chronic disease, poverty, and environmental hazards.^15,16,17^ In many cases, these inequities are due to growing disinvestment in rural communities and policy decisions that bias toward urban areas.^18^ As a consequence, people living in rural areas in the US are at increased risk of poor health outcomes compared with their urban counterparts. For example, rural adults are more likely to die from heart disease, cancer, unintentional injury deaths, stroke, and chronic lower respiratory disease than urban adults, and these gaps have widened in recent years.^15,17^

Structural racism and underinvestment in rural health hospitals and clinics put individuals at risk. Given the impact of intersectionality, minoritized individuals in rural areas may be especially vulnerable to health inequities.^18,19^ This is seen among Black rural adults who have an increased chronic disease burden, decreased health-related quality of life, and decreased health care access and use compared with White rural adults.^19^ Little is known about how SDOH and structural factors are associated with the risk of health care–associated infections (HAIs),^20,21^ which are a critical public health problem in the US. HAIs affect 3% to 4% of hospitalized patients and result in approximately 11% in-hospital mortality.^22,23^ Structural racism and rurality could influence HAIs through a number of mechanisms, including increased susceptibility to infection due to worse underlying health status at admission, interpersonal bias, or differential diagnosis and treatment in health care settings. We sought to evaluate the association of these structural factors as they relate to HAIs and adverse outcomes of HAIs using the proxies of patient race and urban vs rural residence.

## Methods

### Data Source

Data were queried from the BJC Healthcare Informatics database, which contains electronic health record (EHR) data from a large hospital network. We included 3 network hospitals: 1 urban, tertiary care referral hospital and 2 suburban, community hospitals. The EHR data include microbiology and other laboratory results, vital signs, medications, demographics, and billing data.^24^ This study was approved by the Washington University School of Medicine institutional review board with a waiver of informed consent per section 164.512(i) of the Privacy Rule. We followed the Strengthening the Reporting of Observational Studies in Epidemiology (STROBE) reporting guidelines for cohort studies.

### Study Cohort

We assembled a cohort of patients aged 18 years or older who were admitted for 48 hours or longer from January 1, 2017, to August 31, 2020. Patients had to be discharged by September 30, 2020, to allow complete capture of admission information. We excluded psychiatric, rehabilitation, obstetrics and/or gynecology, and hospice admissions owing to the low risk of HAIs. The study was restricted to Black and White patients because of the large proportion of unknown, other, or missing race reported for patients who were not Black or White. For the analysis of the outcome of intensive care unit (ICU) admission among HAI admissions, the cohort was further restricted to those who had not been admitted to the ICU before HAI.

### Race and Rurality Definition

We classified patients on the basis of the combination of 2 SDOH^1^: patient race (Black or White, as recorded in the EHR; see eTable 1 in Supplement 1 for categories) and rurality of the patient’s home (urban or rural). These 2 SDOH were used as proxies to represent structural racism and disinvestment in rural communities.

Rurality was defined according to the 2013 National Center for Health Statistics Urban-Rural Classification Scheme for Counties.^25^ A zip code to county crosswalk was used to convert the patient home zip code from the EHR to the county level.^26^ If a zip code spanned more than 1 county, we assigned it to the county with the highest residential ratio. The multilevel classification was dichotomized into urban (ie, metropolitan counties within metropolitan statistical areas with ≥50 000 population) and rural (ie, nonmetropolitan [micropolitan and noncore] counties). Admissions with a missing zip code or no match to the crosswalk and/or National Center for Health Statistics data were assigned to the urban category (0.02% of the cohort).

### Outcome Definitions

HAIs were defined according to the timing and microbiology of positive cultures from blood, respiratory, and urine specimens across the 3 study hospitals, with each specimen source considered separately. The culture collection date and time was used to determine whether the infection was a community-associated infection, defined as a positive culture obtained less than 48 hours after admission, or an HAI, defined as a positive culture obtained 48 hours or more after admission. Using Centers for Disease Control and Prevention standards,^27^ as we have done previously,^28,29,30^ repeat infections were defined as a positive culture from the same specimen source more than 14 days from the original positive culture. Therefore, a patient with a community-associated infection could develop an HAI at the same site during the same admission according to this repeat infection timeline, but repeat positive cultures from the same site within 14 days were considered a single infection episode. For blood cultures, we excluded common contaminants (eg, diphtheroids and* Micrococcus* species) and excluded coagulase-negative *Staphylococcus* species unless the coagulase-negative *Staphylococcus* species was identified on more than 1 culture date.^27^ For HAIs, a positive blood culture 3 days before or after a positive respiratory or urine culture with a matching organism was considered a secondary infection rather than a blood stream HAI. Adverse outcomes of HAIs during the admission included ICU admission on or after the HAI date and in-hospital death.

### Covariates

Baseline covariates during the admission included 2 additional SDOH of interest: Medicaid insurance and quartile of national household income (lowest income quartile vs other 3 income quartiles). We used patient home zip code to determine the US Census Bureau median household income^31^ and referenced national income thresholds^32^ to determine whether the patient resided in an area corresponding to the lowest national income quartile. Admissions missing a zip code or without a household income data match (0.4% of the cohort) were assigned the median income of the overall cohort.

Additional covariates included patient sex, age group, year of admission, study hospital, comorbidities, body mass index (calculated as weight in kilograms divided by height in meters squared) category, and modified Acute Physiology and Chronic Health Evaluation (APACHE) II score quartile. Comorbidities were defined using *International Statistical Classification of Diseases, Tenth Revision, Clinical Modification* diagnosis codes and the Elixhauser classification.^33,34^ A modified APACHE score was calculated using laboratory results and vital sign measurements in the first 24 hours of the admission including all items except for Glasgow Coma Scale score.

### Statistical Analysis

We summarized the distribution of baseline characteristics within each race and rurality subpopulation. We considered the White urban subpopulation the reference group because it was the largest subpopulation and would, therefore, enhance model estimate stability. Before conducting multivariable analyses, multicollinearity was assessed by examining tolerance values to ensure independence of variables. To determine the association between SDOH-related factors and HAI, we used generalized estimating equations models with a Poisson distribution and log link to estimate adjusted relative risks (aRRs) and 95% CIs, accounting for clustering of admissions within a patient using an exchangeable correlation structure and adjusting for covariates. We modeled composite HAI (defined as a positive blood, respiratory, or urine culture) and separately modeled each source-specific HAI. Among HAI admissions, analogous methods were used to examine outcomes after HAI (ie, ICU admission and in-hospital death).

For the composite HAI, ICU admission, and in-hospital death outcomes, subgroup analyses by hospital type (tertiary and community) were performed. The following sensitivity analyses were also conducted: (1) excluding COVID-19 pandemic era admissions (defined by the first COVID-19 admission date in the data), and (2) restricting to admissions with a nonsurgery admitting service since surgery admissions are more likely to have instrumentation (eg, urine catheter). All analyses were performed from November 2022 to April 2024 using SAS statistical software version 9.4 (SAS Institute). Statistical significance was defined as a 95% CI that did not cross the null value of 1.

## Results

The final cohort included 214 955 patients admitted to the hospital (median [IQR] age, 63 [51-73] years; 108 679 female patients [50.6%]), after exclusions for race other than Black or White (8394 admissions, of which 4388 [52%] were unknown, other, or missing race) and admitting services at low risk of an HAI (31 443 admissions). The final cohort included 71 391 Black urban patients (33.2%), 1099 Black rural patients (0.5%), 108 273 White urban patients (50.4%), and 34 192 White rural patients (15.9%). The Table presents patient characteristics of admissions by race and rurality.

**Table.** Baseline Patient Characteristics by Race and Rurality, Among Adults Admitted for 48 Hours or Longer to 3 US Hospitals

Characteristic	Patients, No. (%)			
	Black		White	
	Urban (n = 71 391)	Rural (n = 1099)	Urban (n = 108 273)	Rural (n = 34 192)
Age, median (IQR), y	59 (46-69)	54 (37-63)	66 (54-76)	63 (52-72)
Sex				
Female	38 421 (53.8)	465 (42.3)	53 664 (49.6)	16 129 (47.2)
Male	32 970 (46.2)	634 (57.7)	54 609 (50.4)	18 063 (52.8)
Body mass index^a^				
<18.5	3638 (5.1)	49 (4.5)	4286 (4.0)	1257 (3.7)
18.5 to <25	20 281 (28.4)	305 (27.8)	30 849 (28.5)	8587 (25.1)
25 to <30	17 379 (24.3)	238 (21.7)	30 848 (28.5)	9808 (28.7)
30 to <35	11 994 (16.8)	198 (18.0)	19 940 (18.4)	7036 (20.6)
35 to <40	7248 (10.2)	164 (14.9)	10 136 (9.4)	3602 (10.5)
≥40	9066 (12.7)	130 (11.8)	8842 (8.2)	3127 (9.1)
Missing	1785 (2.5)	15 (1.4)	3372 (3.1)	775 (2.3)
Acute physiology and chronic health evaluation score quartile				
First	11 692 (16.4)	234 (21.3)	24 546 (22.7)	7906 (23.1)
Second	15 926 (22.3)	253 (23.0)	27 608 (25.5)	8485 (24.8)
Third	21 369 (29.9)	317 (28.8)	31 422 (29.0)	9711 (28.4)
Fourth (highest values)	22 404 (31.4)	295 (26.8)	24 697 (22.8)	8090 (23.7)
Census median household income in lowest national income quartile	47 433 (66.4)	693 (63.1)	15 778 (14.6)	17 627 (51.6)
Medicaid insurance^b^	33 819 (47.4)	611 (55.6)	15 954 (14.7)	8447 (24.7)
Comorbidities				
AIDS	1167 (1.6)	13 (1.2)	435 (0.4)	42 (0.1)
Alcohol abuse	5549 (7.8)	57 (5.2)	5367 (5.0)	1305 (3.8)
Chronic kidney disease	23 635 (33.1)	261 (23.7)	24 017 (22.2)	7551 (22.1)
Chronic pulmonary disease	22 829 (32.0)	245 (22.3)	27 594 (25.5)	9724 (28.4)
Coagulopathy	6277 (8.8)	143 (13.0)	11 594 (10.7)	4287 (12.5)
Congestive heart failure	23 112 (32.4)	330 (30.0)	25 267 (23.3)	8522 (24.9)
Deficiency anemias	23 845 (33.4)	296 (26.9)	23 139 (21.4)	6857 (20.1)
Depression	11 214 (15.7)	196 (17.8)	24 836 (22.9)	7213 (21.1)
Diabetes	29 410 (41.2)	375 (34.1)	31 727 (29.3)	11 033 (32.3)
Drug abuse	5938 (8.3)	73 (6.6)	3282 (3.0)	891 (2.6)
Hypertension	54 705 (76.6)	705 (64.1)	71 711 (66.2)	22 643 (66.2)
Liver disease	5283 (7.4)	89 (8.1)	8973 (8.3)	3323 (9.7)
Lymphoma	1339 (1.9)	31 (2.8)	4219 (3.9)	1389 (4.1)
Metastatic cancer	3573 (5.0)	83 (7.6)	9427 (8.7)	3319 (9.7)
Other neurological disorders	11 329 (15.9)	140 (12.7)	17 843 (16.5)	5030 (14.7)
Paralysis	6995 (9.8)	104 (9.5)	5816 (5.4)	1816 (5.3)
Peripheral vascular disease	6801 (9.5)	87 (7.9)	11 377 (10.5)	4116 (12.0)
Pulmonary circulation disease	1963 (2.7)	53 (4.8)	2556 (2.4)	912 (2.7)
Rheumatoid arthritis and/or collagen vascular disease	3773 (5.3)	52 (4.7)	5699 (5.3)	1537 (4.5)
Solid tumor without metastasis	2845 (4.0)	74 (6.7)	7992 (7.4)	3045 (8.9)
Hospital				
1	39 074 (54.7)	947 (86.2)	63 678 (58.8)	25 648 (75.0)
2	25 703 (36.0)	79 (7.2)	11 915 (11.0)	548 (1.6)
3	6614 (9.3)	73 (6.6)	32 680 (30.2)	7996 (23.4)
Transfer into admission	8262 (11.6)	473 (43.0)	23 021 (21.3)	14 145 (41.4)
Year of admission				
2017	17 433 (24.4)	293 (26.7)	28 006 (25.9)	9428 (27.6)
2018	19 382 (27.1)	311 (28.3)	29 912 (27.6)	9382 (27.4)
2019	20 945 (29.3)	311 (28.3)	31 862 (29.4)	9794 (28.6)
2020	13 631 (19.1)	184 (16.7)	18 493 (17.1)	5588 (16.3)
Community-associated infection				
Blood, respiratory, or urine	6085 (8.5)	72 (6.6)	8570 (7.9)	1910 (5.6)
Blood	2309 (3.2)	41 (3.7)	2733 (2.5)	662 (1.9)
Respiratory	560 (0.8)	8 (0.7)	1161 (1.1)	451 (1.3)
Urine	3839 (5.4)	33 (3.0)	5440 (5.0)	902 (2.6)

^a^ Body mass index is calculated as weight in kilograms divided by height in meters squared.

^b^ Refers to Medicaid insurance, including dual Medicare and Medicaid insurance, compared with all other payer types (ie, Medicare, Veterans Affairs, private insurance, self-pay, or none listed).

### Health Care–Associated Infections

HAIs occurred during 6699 admissions (3.1%) and included 1572 blood infections, 2497 respiratory infections, and 3146 urine infections (they were not mutually exclusive). Among HAI admissions, the total length of stay was a median (IQR) of 19 (11-31) days, and the median (IQR) number of days hospitalized before HAI was 7 (4-13) days (eTable 2 in Supplement 1). HAIs developed during 1955 admissions (2.7%) among Black urban patients, 45 admissions (4.1%) among Black rural patients, 3368 admissions (3.1%) among White urban patients, and 1331 admissions (3.9%) among White rural patients (eFigure 1 in Supplement 1).

**Figure 1.**
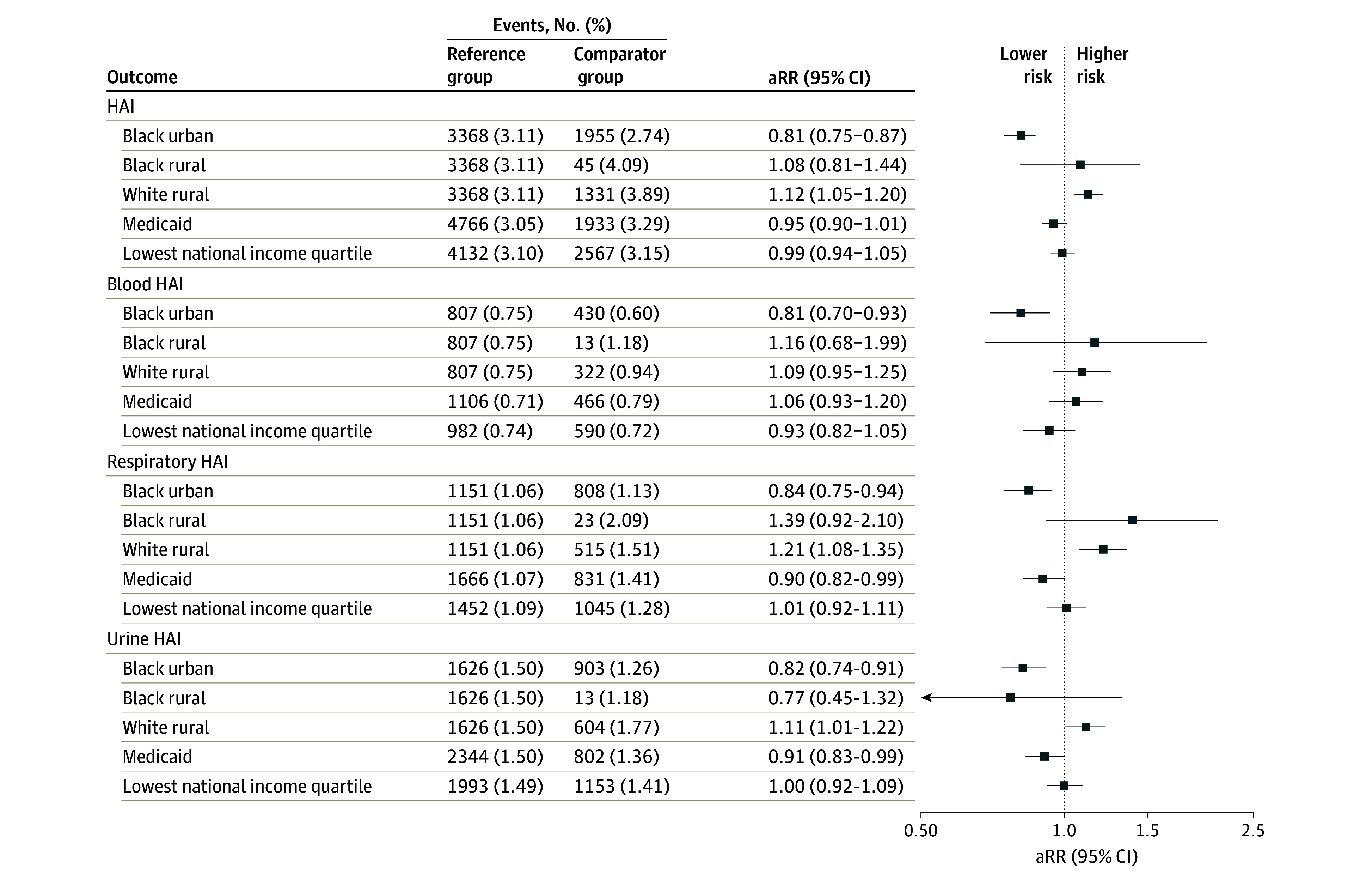
Adjusted Relative Risk (aRR) Estimates for Health Care–Associated Infections (HAIs), Overall and by Specimen Source Each model was adjusted for age group (18-34, 35-54, 55-64, 65-74, and ≥75 years), sex, Acute Physiology and Chronic Health Evaluation score quartile, body mass index category, comorbidities, hospital, and year of admission. Reference group for Black urban, Black rural, and White rural is White urban patients. Reference group for Medicaid is any other payer type. Reference group for lowest national income quartile is the highest 3 income quartiles combined.

eTable 3 in Supplement 1 presents the distribution of HAI and source-specific HAIs by select SDOH (race and rurality, Medicaid insurance, and median household income). Compared with admissions among White urban patients, we observed a decreased risk of HAI among Black urban patients (aRR, 0.81; 95% CI, 0.75-0.87) and an increased risk of HAI among White rural patients (aRR, 1.12; 95% CI, 1.05-1.20), after accounting for potential confounders (Figure 1). Black rural patients had a risk of HAI similar to that of White urban patients (aRR, 1.08; 95% CI, 0.81-1.44). Admissions among patients with Medicaid insurance and among patients from the lowest income neighborhoods had no differential risk of HAI. These findings were generally consistent for blood, respiratory, and urine HAIs, except that White rural patients had risk of blood HAIs similar to that of White urban patients, and Medicaid insurance was associated with decreased risk of respiratory and urine HAIs.

In subgroup analyses, the associations of race and rurality with HAI risk were largely consistent in analyses restricted to the tertiary care hospital and restricted to the community hospitals (eFigure 2 in Supplement 1). Sensitivity analyses restricted to the pre–COVID-19 era and restricted to admissions with a nonsurgical admitting service had results similar to those of the primary analyses, with Black urban patients at decreased risk of HAI (eFigure 2 in Supplement 1).

### ICU Admissions Among HAI Admissions

**Figure 2.**
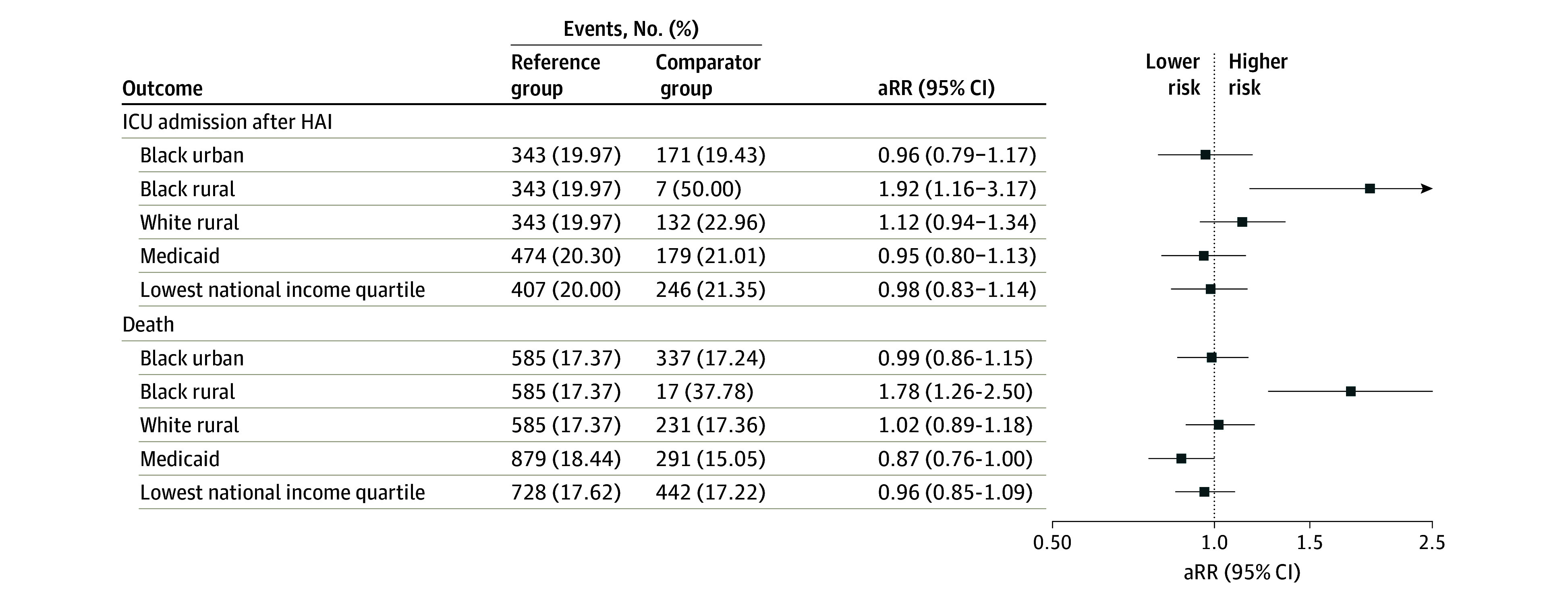
Adjusted Relative Risk (aRR) Estimates for Intensive Care Unit (ICU) Admission and Death Among Health Care–Associated Infection (HAI) Admissions HAI admissions with an ICU admission before the HAI date were excluded from the ICU admission outcome model. Each model was adjusted for age group (18-34, 35-54, 55-64, 65-74, and ≥75 years), sex, Acute Physiology and Chronic Health Evaluation score quartile, body mass index category, comorbidities, hospital, and year of admission. Reference group for Black urban, Black rural, and White rural is White urban patients. Reference group for Medicaid is any other payer type. Reference group for lowest national income quartile is the highest 3 income quartiles combined.

Among 3187 HAI admissions without prior ICU during the admission, 653 (20.5%) resulted in an ICU admission after HAI; the proportion was much higher among Black rural patients (7 admissions [50.0%]) compared with Black urban patients (171 admissions [19.4%]), White urban patients (343 admissions [20.0%]), and White rural patients (132 admissions [23.0%]) (eFigure 3 in Supplement 1). eTable 4 in Supplement 1 presents the distribution of ICU admission by SDOH among HAI admissions without prior ICU care. In multivariable analysis, compared with White urban patients, Black rural patients had an increased risk of ICU admission (aRR, 1.92; 95% CI, 1.16-3.17), whereas Black urban patients (aRR, 0.96; 95% CI, 0.79-1.17) and White rural patients (aRR, 1.12; 95% CI, 0.94-1.34) had outcomes similar to those of White urban patients (Figure 2). Medicaid insurance and neighborhood income quartile were not associated with ICU admission. The directionality of sensitivity and subgroup analyses was consistent with the main results, with Black rural patients at increased risk of ICU admission, however the estimates were imprecise with confidence intervals spanning the null for some analyses (eFigure 4 in Supplement 1).

### Death Among HAI Admissions

A total of 1170 patients with HAI (17.5%) died; the proportion of admissions resulting in death was 37.8% (17 patients) among Black rural patients, 17.2% (337 patients) among Black urban patients, 17.4% (585 patients) among White urban patients, and 17.4% (231 patients) among White rural patients (eFigure 3 in Supplement 1). eTable 4 in Supplement 1 presents the distribution of death by SDOH. In multivariable analysis, Black rural patients had an increased risk of in-hospital death (aRR, 1.78; 95% CI 1.26-2.50), whereas White rural patients (aRR, 1.02; 95% CI, 0.89-1.18) and Black urban patients (aRR, 0.99; 95% CI, 0.86-1.15) had outcomes similar to those of White urban patients (Figure 2). There were no significant differences in mortality by neighborhood income quartile or Medicaid insurance. Sensitivity and subgroup analyses were consistent with the main results with Black rural patients at increased risk of death (eFigure 5 in Supplement 1).

## Discussion

In this cohort study of hospitalized patients, we demonstrated inequities in risk of HAIs and HAI outcomes. Compared with White urban patients, Black urban patients had a lower risk of HAI, whereas White rural patients had an increased risk of HAI. However, among patients with an HAI, Black rural patients had markedly higher risk of mortality, even after accounting for potential confounders. Collectively, these findings suggest that SDOH may contribute to patient outcomes through structural mechanisms linked to racism, rural disinvestment, and care delivery.

Black urban patients had a decreased risk of HAI compared with White urban patients, which is counter to prior studies.^21,35,36,37,38^ However, although Black urban patients were at decreased risk of acquiring an HAI, they were relatively sicker, with higher APACHE scores and higher burden of comorbidities, and were more likely to have a positive culture than White urban patients in the first 48 hours of admission. If Black urban patients were more likely to be treated with antibiotics upon arrival because of their community-associated infection, that could explain their lower risk of HAI. White rural patients had an increased risk of HAI compared with White urban patients. As discussed, rural residence is likely serving as a marker for poor health determinants that lead to higher risk of infection out of the hospital, as well as higher susceptibility to infection once in the hospital.

Among patients with an HAI, Black rural patients were at markedly increased risk of ICU admission and death compared with White urban patients, even after adjusting for factors including comorbidities and measures of poverty. One possible explanation could be differences in treatment during hospitalization, as prior studies^39,40^ have documented that hospitalized Black patients are more likely to receive non–first-line antibiotic treatments. It is also possible that Black rural patients are sicker in ways that we could not measure in our data owing to the confluence of inequities related to racism and rural residence. On a national level, the worst health outcomes for cardiovascular disease, cancer, diabetes, and many other conditions are seen at the confluence of race, rurality, and poverty; these markers may translate into a poorer ability to tolerate or compensate for an HAI.^41,42,43,44,45^

At urban and suburban hospitals, and particularly referral centers, it is important for clinicians to be mindful of the potential impact of race and rurality to help mitigate risk of HAIs and subsequent adverse outcomes. Although race and rurality are not biological constructs, they are important proxies for structural disadvantage, reflecting undermeasured or unmeasured social risk.^46^ As such, they could serve as important markers for individuals who should receive additional clinical attention to reduce adverse events. Developing interventions specifically aimed at improving outcomes for Black and rural patients is crucial as we seek to reduce health inequities related to these structural and systemic factors.

### Limitations

Our study has several possible limitations. First, our results are subject to possible residual confounding. We attempted to mitigate confounding by using regression models that accounted for a variety of patient characteristics, including granular patient data available in the EHR (eg, modified APACHE score and body mass index). However, not all SDOH are accurately measurable using EHR or linked data sources.^47^ Second, the potential for selection bias exists since our study population is drawn from suburban and urban hospitals; it is possible that only the sickest rural patients were included in our cohort because they were more likely than urban patients to be transferred to urban hospitals. However, we found that Black rural patients, but not White rural patients, had increased risk of adverse outcomes after HAI and adjusted for severity of illness (via modified APACHE score).

Third, the interpretation of our results is limited by the validity of the HAI outcome definition. A strength of our study was the use of culture data rather than *International Statistical Classification of Diseases, Tenth Revision, Clinical Modification* diagnosis codes to define HAI; however, we acknowledge that not all positive cultures represent true infection. Yet, in a previous analysis^28^ examining risk factors for HAI, we performed sensitivity analyses restricting HAI to a positive culture with an associated infection-related diagnosis code and had results similar to the primary definition based solely on a positive culture. Fourth, Black rural patients represented the smallest subpopulation, which resulted in larger confidence intervals in our models compared with other subpopulations and precluded some subgroup analyses, including the ability to examine outcomes for source-specific HAI. It is noted that Black rural patients had a higher proportion of respiratory HAIs than other race and rurality groups, which may be associated with higher mortality than other HAIs; future studies with larger rural populations are warranted. Fifth, our findings may have limited generalizability because our study was restricted to patients at nonrural hospitals from a single health care system. Nevertheless, this is an important population to study as more care for rural patients is now provided in urban settings due to the large increase in closures of critical access hospitals and rural hospitals.^48^

## Conclusions

In this cohort study of hospitalized adults, we identified inequities related to race and rurality in HAIs and adverse outcomes from HAIs, likely reflecting the greater burden of structural and systemic barriers to health faced by Black individuals and rural residents. Our results also highlight the importance of considering the intersection of these 2 types of structural risk factors—race and rurality. By identifying populations vulnerable to HAIs and negative outcomes, we lay the groundwork for additional health equity studies to further investigate the relationships underpinning these inequities and develop comprehensive solutions to achieve health equity.^49^ Targeting structural factors through policies addressing health care and beyond is needed to ultimately close gaps in health equity.

## Notes

Supplement 1. **eFigure 1.** Proportion of admissions with a healthcare-associated infection (HAI) by race and rurality **eFigure 2.** Adjusted relative risk estimates for healthcare-associated infection (HAI), sensitivity/subgroup analyses **eFigure 3.** Among healthcare-associated infections (HAIs), proportion of admissions with adverse outcomes by race and rurality **eFigure 4.** Adjusted relative risk estimates for intensive care unit admission among HAI admissions, sensitivity/subgroup analyses **eFigure 5.** Adjusted relative risk estimates for death among HAI admissions, sensitivity/subgroup analyses **eTable 1.** Race categories as reported in the electronic health record for inpatient admissions during the study period **eTable 2.** Characteristics of healthcare-associated infections (HAIs) **eTable 3.** Social determinants of health and healthcare-associated infections (HAIs) **eTable 4.** Social determinants of health and adverse outcomes among healthcare-associated infection admissions

Supplement 2.
Data Sharing Statement

## References

[1] US Department of Health and Human Services Office of Disease Prevention and Health Promotion. Healthy People 2030: social determinants of health. Accessed December 14, 2023. https://health.gov/healthypeople/priority-areas/social-determinants-health

[2] Centers for Disease Control and Prevention. Social determinants of health. May 15, 2024. Accessed September 10, 2024. https://www.cdc.gov/public-health-gateway/php/about/social-determinants-of-health.html

[3] Yearby R, Clark B, Figueroa JF. Structural racism in historical and modern US health care policy. Health Aff (Millwood). 2022;41(2):187-194. doi:10.1377/hlthaff.2021.01466 35130059

[4] Yearby R. Structural racism and health disparities: reconfiguring the social determinants of health framework to include the root cause. J Law Med Ethics. 2020;48(3):518-526. doi:10.1177/1073110520958876 33021164

[5] Baciu A, Negussie Y, Geller A, Weinstein JN, eds. Communities in Action: Pathways to Health Equity. National Academies Press; 2017. doi:10.17226/24624 28418632

[6] Bailey ZD, Feldman JM, Bassett MT. How structural racism works: racist policies as a root cause of U.S. racial health inequities. N Engl J Med. 2021;384(8):768-773. doi:10.1056/NEJMms2025396 33326717 PMC11393777

[7] Churchwell K, Elkind MSV, Benjamin RM, ; American Heart Association. Call to action: structural racism as a fundamental driver of health disparities—a presidential advisory from the American Heart Association. Circulation. 2020;142(24):e454-e468. doi:10.1161/CIR.0000000000000936 33170755

[8] Caraballo C, Ndumele CD, Roy B, . Trends in racial and ethnic disparities in barriers to timely medical care among adults in the US, 1999 to 2018. JAMA Health Forum. 2022;3(10):e223856. doi:10.1001/jamahealthforum.2022.3856 36306118 PMC9617175

[9] Mahajan S, Caraballo C, Lu Y, . Trends in differences in health status and health care access and affordability by race and ethnicity in the United States, 1999-2018. JAMA. 2021;326(7):637-648. doi:10.1001/jama.2021.9907 34402830 PMC8371573

[10] Johnston KJ, Hammond G, Meyers DJ, Joynt Maddox KE. Association of race and ethnicity and Medicare program type with ambulatory care access and quality measures. JAMA. 2021;326(7):628-636. doi:10.1001/jama.2021.10413 34402828 PMC8371568

[11] Bailey ZD, Krieger N, Agénor M, Graves J, Linos N, Bassett MT. Structural racism and health inequities in the USA: evidence and interventions. Lancet. 2017;389(10077):1453-1463. doi:10.1016/S0140-6736(17)30569-X 28402827

[12] Sheehy S, Aparicio HJ, Palmer JR, . Perceived interpersonal racism and incident stroke among US Black women. JAMA Netw Open. 2023;6(11):e2343203. doi:10.1001/jamanetworkopen.2023.43203 37948073 PMC10638652

[13] Javed Z, Haisum Maqsood M, Yahya T, . Race, racism, and cardiovascular health: applying a social determinants of health framework to racial/ethnic disparities in cardiovascular disease. Circ Cardiovasc Qual Outcomes. 2022;15(1):e007917. doi:10.1161/CIRCOUTCOMES.121.007917 35041484

[14] Sistrunk C, Tolbert N, Sanchez-Pino MD, . Impact of federal, state, and local housing policies on disparities in cardiovascular disease in Black/African American men and women: from policy to pathways to biology. Front Cardiovasc Med. 2022;9:756734. doi:10.3389/fcvm.2022.756734 35509276 PMC9058117

[15] Centers for Disease Control and Prevention. About rural health. Accessed October 6, 2023. https://www.cdc.gov/rural-health/php/about/?CDC_AAref_Val=https://www.cdc.gov/ruralhealth/about.html

[16] Caldwell JT, Ford CL, Wallace SP, Wang MC, Takahashi LM. Intersection of living in a rural versus urban area and race/ethnicity in explaining access to health care in the United States. Am J Public Health. 2016;106(8):1463-1469. doi:10.2105/AJPH.2016.303212 27310341 PMC4940644

[17] Harrington RA, Califf RM, Balamurugan A, . Call to action: rural health—a presidential advisory from the American Heart Association and American Stroke Association. Circulation. 2020;141(10):e615-e644. doi:10.1161/CIR.0000000000000753 32078375

[18] Sosin AN, Carpenter-Song EA. Reimagining rural health equity: understanding disparities and orienting policy, practice, and research in rural America. Health Aff (Millwood). 2024;43(6):791-797. doi:10.1377/hlthaff.2024.00036 38830148

[19] James CV, Moonesinghe R, Wilson-Frederick SM, Hall JE, Penman-Aguilar A, Bouye K. Racial/ethnic health disparities among rural adults—United States, 2012-2015. MMWR Surveill Summ. 2017;66(23):1-9. doi:10.15585/mmwr.ss6623a1 29145359 PMC5829953

[20] McGrath CL, Logan LK, Deloney VM, . Monitoring health disparities in healthcare-associated infection surveillance: a Society for Healthcare Epidemiology of America (SHEA) Research Network (SRN) Survey. Infect Control Hosp Epidemiol. 2024;45(4):526-529. doi:10.1017/ice.2023.181 37700531 PMC11007321

[21] Chen J, Khazanchi R, Bearman G, Marcelin JR. Racial/ethnic inequities in healthcare-associated infections under the shadow of structural racism: narrative review and call to action. Curr Infect Dis Rep. 2021;23(10):17. doi:10.1007/s11908-021-00758-x 34466126 PMC8390539

[22] Magill SS, O’Leary E, Janelle SJ, ; Emerging Infections Program Hospital Prevalence Survey Team. Changes in prevalence of health care-associated infections in U.S. hospitals. N Engl J Med. 2018;379(18):1732-1744. doi:10.1056/NEJMoa1801550 30380384 PMC7978499

[23] Magill SS, Edwards JR, Bamberg W, ; Emerging Infections Program Healthcare-Associated Infections and Antimicrobial Use Prevalence Survey Team. Multistate point-prevalence survey of health care-associated infections. N Engl J Med. 2014;370(13):1198-1208. doi:10.1056/NEJMoa1306801 24670166 PMC4648343

[24] Doherty J, Noirot LA, Mayfield J, . Implementing GermWatcher, an enterprise infection control application. AMIA Annu Symp Proc. 2006;2006:209-213. 17238333 PMC1839697

[25] Centers for Disease Control and Prevention. NCHS urban-rural classification scheme for counties. Accessed February 5, 2023. https://www.cdc.gov/nchs/data-analysis-tools/urban-rural.html

[26] Office of Policy Development and Research. HUD USPS ZIP code crosswalk files. Accessed November 26, 2022. https://www.huduser.gov/portal/datasets/usps_crosswalk.html

[27] Centers for Disease Control and Prevention. Identifying healthcare-associated infections (HAI) for NHSN surveillance. Accessed January 31, 2021. https://www.cdc.gov/nhsn/pdfs/pscmanual/2psc_identifyinghais_nhsncurrent.pdf

[28] Kwon JH, Nickel KB, Reske KA, . Risk factors for hospital-acquired infection during the SARS-CoV-2 pandemic. J Hosp Infect. 2023;133:8-14. doi:10.1016/j.jhin.2022.11.020 36493966 PMC9724556

[29] Sahrmann JM, Nickel KB, Stwalley D, . Healthcare-associated infections (HAIs) during the coronavirus disease 2019 (COVID-19) pandemic: a time-series analysis. Antimicrob Steward Healthc Epidemiol. 2023;3(1):e14. doi:10.1017/ash.2022.361 36714284 PMC9879893

[30] Gandra S, Alvarez-Uria G, Stwalley D, . Microbiology clinical culture diagnostic yields and antimicrobial resistance proportions before and during the COVID-19 pandemic in an Indian community hospital and two US community hospitals. Antibiotics (Basel). 2023;12(3):537. doi:10.3390/antibiotics12030537 36978404 PMC10044523

[31] US Census Bureau. Median household income, past 12 months (in 2019 Inflation-Adjusted Dollars). Source: U.S. Census Bureau, 2015-2019 American Community Survey 5-year estimates. Accessed January 31, 2021. https://data.census.gov/cedsci/

[32] Healthcare Cost and Utilization Project (HCUP); Agency for Healthcare Research and Quality. NIS description of data elements. Accessed September 25, 2023. https://hcup-us.ahrq.gov/db/vars/zipinc_qrtl/nisnote.jsp

[33] Elixhauser A, Steiner C, Harris DR, Coffey RM. Comorbidity measures for use with administrative data. Med Care. 1998;36(1):8-27. doi:10.1097/00005650-199801000-00004 9431328

[34] Healthcare Cost and Utilization Project (HCUP); Agency for Healthcare Research and Quality. HCUP Elixhauser comorbidity software. Accessed December 21, 2020. https://hcup-us.ahrq.gov/toolssoftware/comorbidity/comorbidity.jsp

[35] Metersky ML, Hunt DR, Kliman R, . Racial disparities in the frequency of patient safety events: results from the National Medicare Patient Safety Monitoring System. Med Care. 2011;49(5):504-510. doi:10.1097/MLR.0b013e31820fc218 21494115

[36] Jeon CY, Muennig P, Furuya EY, Cohen B, Nash D, Larson EL. Burden of present-on-admission infections and health care-associated infections, by race and ethnicity. Am J Infect Control. 2014;42(12):1296-1302. doi:10.1016/j.ajic.2014.08.019 25465260 PMC4255287

[37] Office of the Assistant Secretary for Planning and Evaluation United States Department of Health and Human Services. Appendices for Report to Congress: social risk factors and performance under Medicare’s value-based payment programs. December 2016. Accessed December 4, 2024. https://aspe.hhs.gov/sites/default/files/migrated_legacy_files//170716/RTCAppendices.pdf

[38] Gettler EB, Kalu IC, Okeke NL, . Disparities in central line-associated bloodstream infection and catheter-associated urinary tract infection rates: an exploratory analysis. Infect Control Hosp Epidemiol. 2023;44(11):1857-1860. doi:10.1017/ice.2023.63 37057848 PMC10665875

[39] Wurcel AG, Essien UR, Ortiz C, . Variation by race in antibiotics prescribed for hospitalized patients with skin and soft tissue infections. JAMA Netw Open. 2021;4(12):e2140798. doi:10.1001/jamanetworkopen.2021.40798 34940871 PMC8703249

[40] Kim C, Kabbani S, Dube WC, . Health equity and antibiotic prescribing in the United States: a systematic scoping review. Open Forum Infect Dis. 2023;10(9):ofad440. doi:10.1093/ofid/ofad440 37671088 PMC10475752

[41] Villapiano N, Iwashyna TJ, Davis MM. Worsening rural-urban gap in hospital mortality. J Am Board Fam Med. 2017;30(6):816-823. doi:10.3122/jabfm.2017.06.170137 29180557

[42] Hammond G, Luke AA, Elson L, Towfighi A, Joynt Maddox KE. Urban-rural inequities in acute stroke care and in-hospital mortality. Stroke. 2020;51(7):2131-2138. doi:10.1161/STROKEAHA.120.029318 32833593

[43] Hammond G, Waken RJ, Johnson DY, Towfighi A, Joynt Maddox KE. Racial inequities across rural strata in acute stroke care and in-hospital mortality: national trends over 6 years. Stroke. 2022;53(5):1711-1719. doi:10.1161/STROKEAHA.121.035006 35172607 PMC9324215

[44] Feigin VL, Vos T, Alahdab F, ; GBD 2017 US Neurological Disorders Collaborators. Burden of neurological disorders across the US From 1990-2017: a global burden of disease study. JAMA Neurol. 2021;78(2):165-176. doi:10.1001/jamaneurol.2020.4152 33136137 PMC7607495

[45] Roth GA, Dwyer-Lindgren L, Bertozzi-Villa A, . Trends and patterns of geographic variation in cardiovascular mortality among US counties, 1980-2014. JAMA. 2017;317(19):1976-1992. doi:10.1001/jama.2017.4150 28510678 PMC5598768

[46] Office of the Assistant Secretary for Planning and Evaluation United States Department of Health and Human Services. Report to Congress: social risk factors and performance under Medicare’s value-based payment programs. December 2016. Accessed December 4, 2024. https://aspe.hhs.gov/sites/default/files/migrated_legacy_files/171041/ASPESESRTCfull.pdf

[47] Bilheimer LT, Klein RJ. Data and measurement issues in the analysis of health disparities. Health Serv Res. 2010;45(5 Pt 2):1489-1507. doi:10.1111/j.1475-6773.2010.01143.x 21054368 PMC2965888

[48] American Hospital Association. Rural hospital closures threaten access: solutions to preserve care in local communities. September 2022. Accessed December 4, 2024. https://www.aha.org/system/files/media/file/2022/09/rural-hospital-closures-threaten-access-report.pdf

[49] Thomas SB, Quinn SC, Butler J, Fryer CS, Garza MA. Toward a fourth generation of disparities research to achieve health equity. Annu Rev Public Health. 2011;32:399-416. doi:10.1146/annurev-publhealth-031210-101136 21219164 PMC3419584

