# Supplementary material for: Intersection of Race and Rurality With Health Care–Associated Infections and Subsequent Outcomes
Source: JAMA Netw Open. 2025 Feb 3;8(2):e2453993. doi: 10.1001/jamanetworkopen.2024.53993 (PMC11791699; doi:10.1001/jamanetworkopen.2024.53993)
Supplement: Supplement 1. — eFigure 1. Proportion of admissions with a healthcare-associated infection (HAI) by race and rurality eFigure 2. Adjusted relative risk estimates for healthcare-associated infection (HAI), sensitivity/subgroup analyses eFigure 3. Among healthcare-associated infections (HAIs), proportion of admissions with adverse outcomes by race and rurality eFigure 4. Adjusted relative risk estimates for intensive care unit admission among HAI admissions, sensitivity/subgroup analyses eFigure 5. Adjusted relative risk estimates for death among HAI admissions, sensitivity/subgroup analyses eTable 1. Race categories as reported in the electronic health record for inpatient admissions during the study period eTable 2. Characteristics of healthcare-associated infections (HAIs) eTable 3. Social determinants of health and healthcare-associated infections (HAIs) eTable 4. Social determinants of health and adverse outcomes among healthcare-associated infection admissions [file jamanetwopen-e2453993-s001.pdf]

Supplemental Online Content

Nickel KB, Kinzer H, Butler AM, et al. Intersection of race and rurality with health care–associated infections and subsequent outcomes. *JAMA Netw Open*. 2025;8(1):e2453993. doi:10.1001/jamanetworkopen.2024.53993

eFigure 1. Proportion of admissions with a healthcare-associated infection (HAI) by race and rurality

eFigure 2. Adjusted relative risk estimates for healthcare-associated infection (HAI), sensitivity/subgroup analyses

eFigure 3. Among healthcare-associated infections (HAIs), proportion of admissions with adverse outcomes by race and rurality

eFigure 4. Adjusted relative risk estimates for intensive care unit admission among HAI admissions, sensitivity/subgroup analyses

eFigure 5. Adjusted relative risk estimates for death among HAI admissions, sensitivity/subgroup analyses

eTable 1. Race categories as reported in the electronic health record for inpatient admissions during the study period

eTable 2. Characteristics of healthcare-associated infections (HAIs)

eTable 3. Social determinants of health and healthcare-associated infections (HAIs)

eTable 4. Social determinants of health and adverse outcomes among healthcare-associated infection admissions

This supplemental material has been provided by the authors to give readers additional information about their work.

© 2025 Nickel KB et al. *JAMA Network Open*.

eFigure 1. Proportion of admissions with a healthcare-associated infection (HAI) by race and rurality

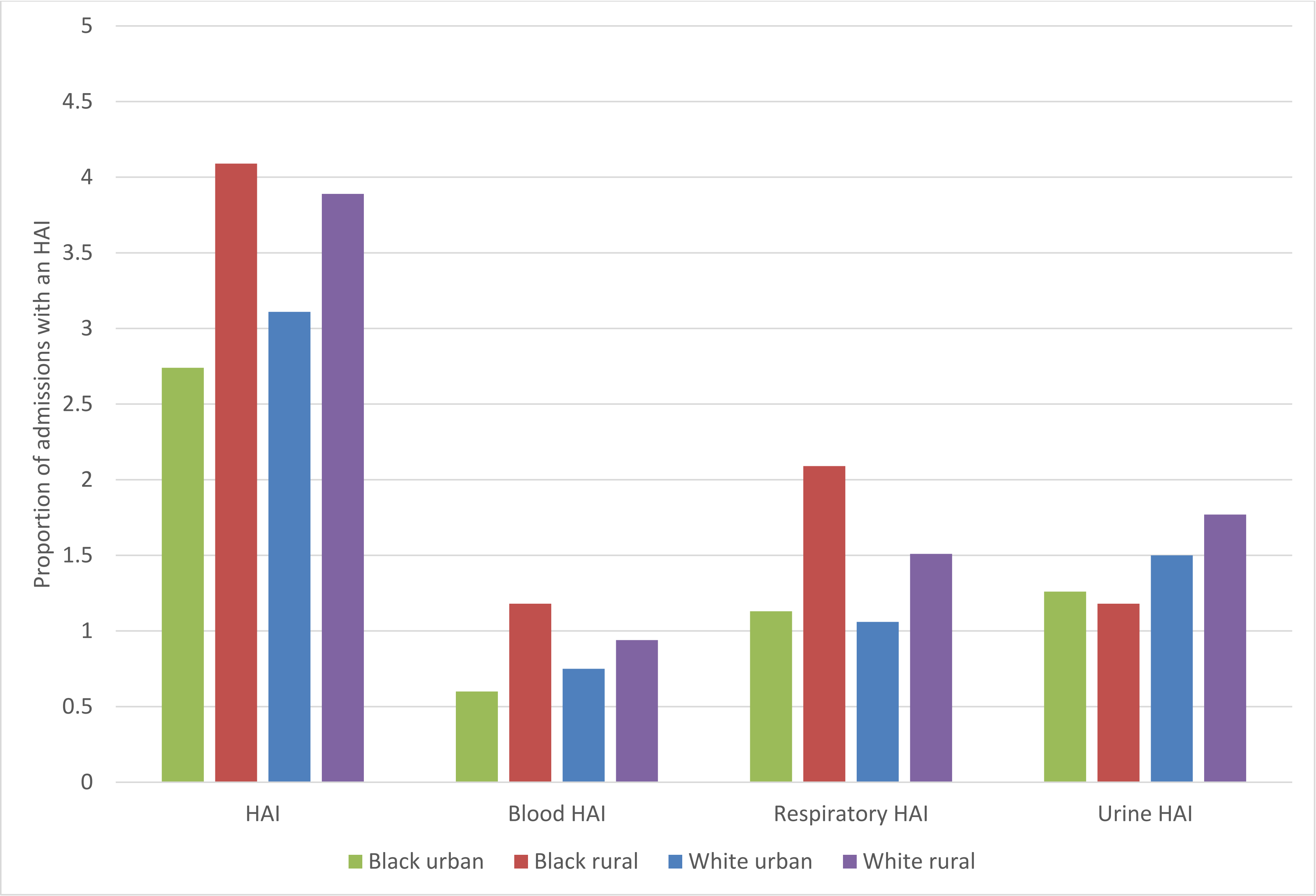

© 2025 Nickel KB et al. *JAMA Network Open*.

eFigure 2. Adjusted relative risk estimates for healthcare-associated infection (HAI), sensitivity/subgroup analyses[a, b]

Outcome	No. of events (%) Reference group	No. of events (%) Comparator group	Adjusted RR (95% CI)
**HAI**			
Black urban	3,368 (3.11)	1,955 (2.74)	0.81 (0.75-0.87)
Black rural	3,368 (3.11)	45 (4.09)	1.08 (0.81-1.44)
White rural	3,368 (3.11)	1,331 (3.89)	1.12 (1.05-1.20)
**Pre-COVID era HAI**			
Black urban	2,946 (3.06)	1,627 (2.62)	0.78 (0.73-0.84)
Black rural	2,946 (3.06)	40 (4.12)	1.11 (0.82-1.50)
White rural	2,946 (3.06)	1,182 (3.87)	1.12 (1.04-1.20)
**Tertiary care hospital HAI**			
Black urban	2,355 (3.70)	1,290 (3.30)	0.82 (0.76-0.89)
Black rural	2,355 (3.70)	41 (4.33)	1.06 (0.79-1.44)
White rural	2,355 (3.70)	1,115 (4.35)	1.14 (1.06-1.23)
**Community hospital HAI**			
Black urban	1,013 (2.27)	665 (2.06)	0.75 (0.67-0.84)
Black rural	1,013 (2.27)	4 (2.63)	NE
White rural	1,013 (2.27)	216 (2.53)	1.07 (0.92-1.25)
**Non-surgery admitting service HAI**			
Black urban	2,396 (3.38)	1,552 (2.62)	0.73 (0.68-0.80)
Black rural	2,396 (3.38)	36 (4.83)	1.24 (0.90-1.71)
White rural	2,396 (3.38)	832 (4.21)	1.10 (1.01-1.20)

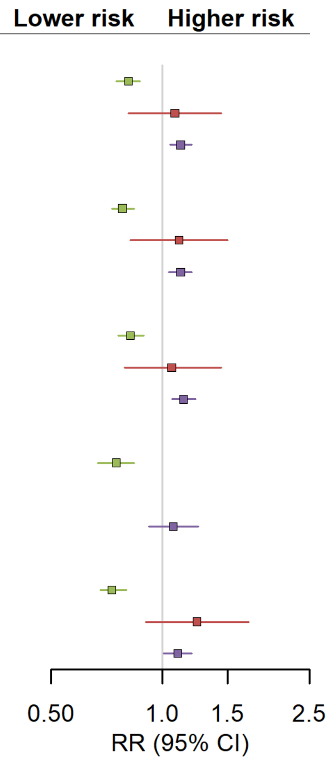

Abbreviations. CI, confidence interval; NE, not estimable; RR, relative risk.

[a] Each model adjusted for age group (18-34 years, 35-54 years, 55-64 years, 65-74 years, ≥ 75 years), sex, APACHE score quartile, BMI category, comorbidities, hospital, year of admission, Medicaid insurance, lowest national income quartile. Reference group for Black urban, Black rural, and White rural is White urban patients. Relative risks were not reported when event counts were less than 5.

[b] Cohort sizes relative to the primary cohort are as follows: pre-COVID era (88.3%), tertiary care hospital (60.2%), community hospitals (39.8%), non-surgical admitting service (70.1%).

© 2025 Nickel KB et al. *JAMA Network Open*.

eFigure 3. Among healthcare-associated infections (HAIs), proportion of admissions with adverse outcomes by race and rurality[a]

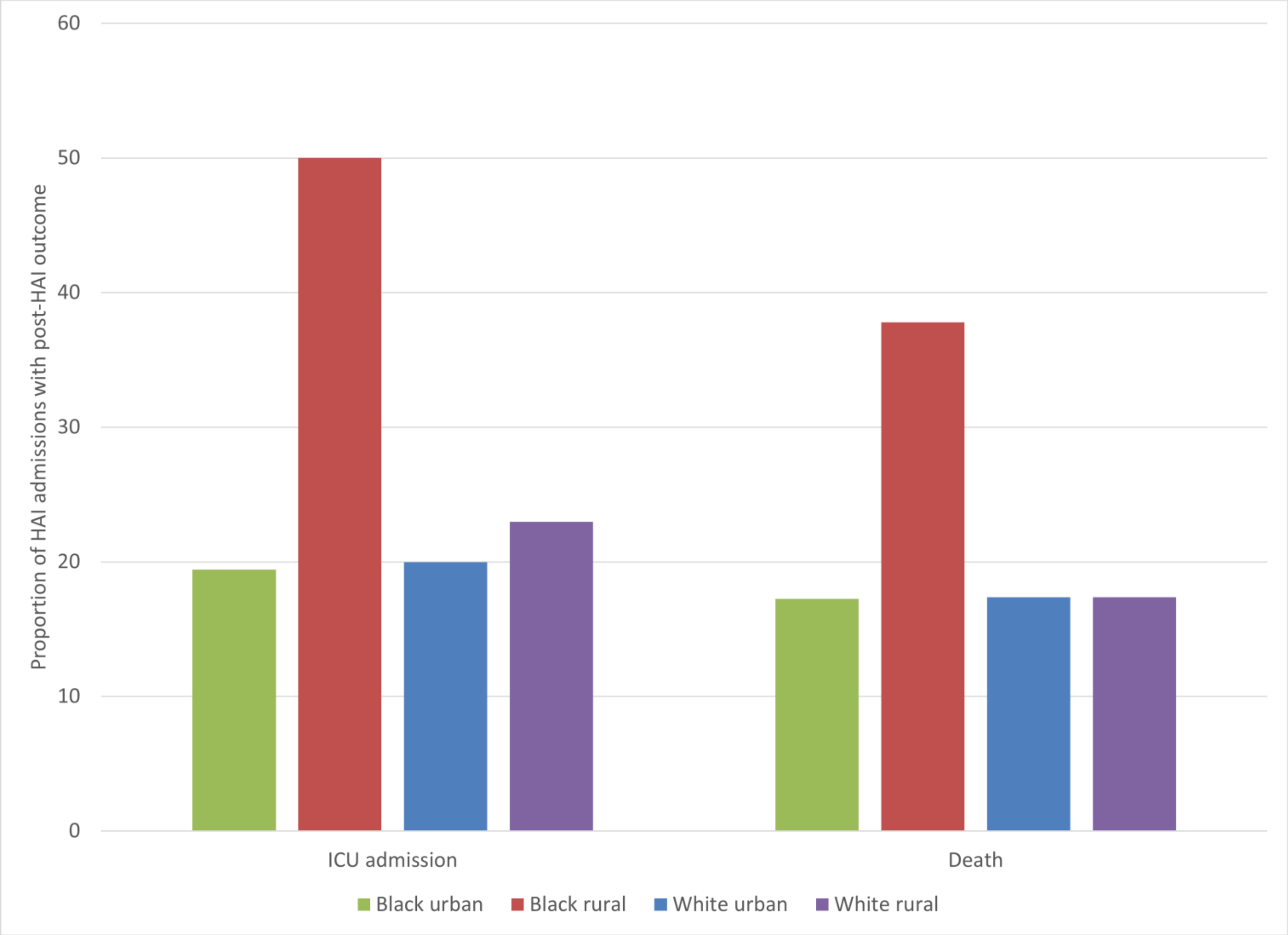

Abbreviations. ICU, intensive care unit.

[a] HAI admissions with an ICU admission before the HAI date were excluded from the post-HAI ICU comparison.

© 2025 Nickel KB et al. *JAMA Network Open*.

eFigure 4. Adjusted relative risk estimates for intensive care unit admission among HAI admissions, sensitivity/subgroup analyses[a]

Outcome	No. of events (%) Reference group	Comparator group	Adjusted RR (95% CI)
**ICU**			
Black urban	343 (19.97)	171 (19.43)	0.96 (0.79-1.17)
Black rural	343 (19.97)	7 (50.00)	1.92 (1.16-3.17)
White rural	343 (19.97)	132 (22.96)	1.12 (0.94-1.34)
**Pre-COVID era ICU**			
Black urban	303 (19.87)	138 (18.04)	0.90 (0.72-1.12)
Black rural	303 (19.87)	6 (50.00)	2.20 (1.31-3.68)
White rural	303 (19.87)	118 (23.23)	1.13 (0.93-1.36)
**Tertiary care hospital ICU**			
Black urban	262 (23.69)	108 (22.09)	0.86 (0.68-1.08)
Black rural	262 (23.69)	5 (41.67)	1.49 (0.82-2.69)
White rural	262 (23.69)	122 (25.52)	1.09 (0.91-1.32)
**Community hospital ICU**			
Black urban	81 (13.24)	63 (16.11)	1.17 (0.79-1.74)
Black rural	81 (13.24)	2 (100.00)	NE
White rural	81 (13.24)	10 (10.31)	0.86 (0.47-1.57)
**Non-surgery admitting service ICU**			
Black urban	300 (21.63)	153 (19.44)	0.90 (0.73-1.11)
Black rural	300 (21.63)	6 (46.15)	1.73 (1.00-3.00)
White rural	300 (21.63)	95 (22.41)	1.01 (0.83-1.24)

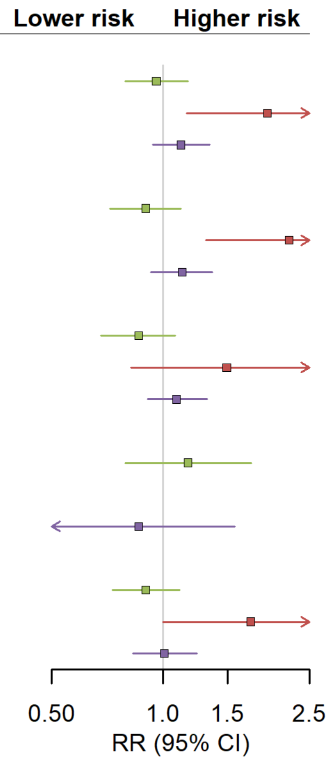

Abbreviations. aRR, adjusted relative risk; CI, confidence interval; NE, not estimable.

[a] HAI admissions with an ICU admission before the HAI date were excluded. Each model adjusted for age group (18-34 years, 35-54 years, 55-64 years, 65-74 years, ≥ 75 years), sex, APACHE score quartile, BMI category, comorbidities, hospital, year of admission, Medicaid insurance, lowest national income quartile. Reference group for Black urban, Black rural, and White rural is White urban patients. Relative risks were not reported when event counts were less than 5.

© 2025 Nickel KB et al. *JAMA Network Open*.

eFigure 5. Adjusted relative risk estimates for death among HAI admissions, sensitivity/subgroup analyses[a]

Outcome	No. of events (%) Reference group	No. of events (%) Comparator group	Adjusted RR (95% CI)
**Death**			
Black urban	585 (17.37)	337 (17.24)	0.99 (0.86-1.15)
Black rural	585 (17.37)	17 (37.78)	1.78 (1.26-2.50)
White rural	585 (17.37)	231 (17.36)	1.02 (0.89-1.18)
**Pre-COVID era death**			
Black urban	495 (16.80)	258 (15.86)	0.93 (0.79-1.10)
Black rural	495 (16.80)	14 (35.00)	1.71 (1.15-2.54)
White rural	495 (16.80)	207 (17.51)	1.05 (0.90-1.23)
**Tertiary care hospital death**			
Black urban	426 (18.09)	206 (15.97)	0.93 (0.78-1.11)
Black rural	426 (18.09)	16 (39.02)	1.69 (1.18-2.43)
White rural	426 (18.09)	191 (17.13)	0.97 (0.82-1.14)
**Community hospital death**			
Black urban	159 (15.70)	131 (19.70)	1.12 (0.87-1.44)
Black rural	159 (15.70)	1 (25.00)	NE
White rural	159 (15.70)	40 (18.52)	1.18 (0.86-1.60)
**Non-surgery admitting service death**			
Black urban	462 (19.28)	292 (18.81)	0.93 (0.79-1.10)
Black rural	462 (19.28)	14 (38.89)	1.71 (1.17-2.50)
White rural	462 (19.28)	157 (18.87)	0.97 (0.82-1.15)

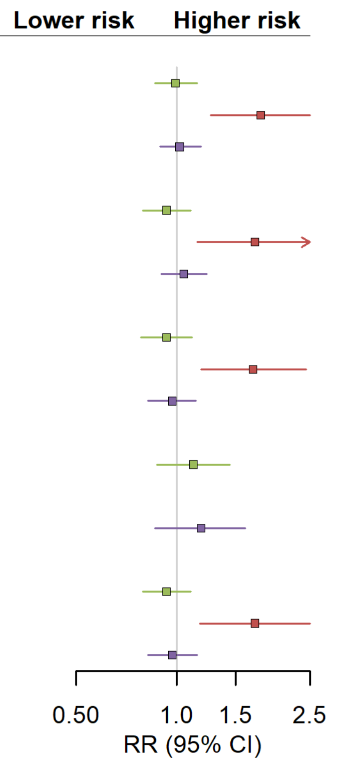

Abbreviations. aRR, adjusted relative risk; CI, confidence interval; NE, not estimable.

[a] Each model adjusted for age group (18-34 years, 35-54 years, 55-64 years, 65-74 years, ≥ 75 years), sex, APACHE score quartile, BMI category, comorbidities, hospital, year of admission, Medicaid insurance, lowest national income quartile. Reference group for Black urban, Black rural, and White rural is White urban patients. Relative risks were not reported when event counts were less than 5.

© 2025 Nickel KB et al. *JAMA Network Open*.

eTable 1. Race categories as reported in the electronic health record for inpatient admissions during the study period

Race category
Alaska Native-Asian
Alaska Native-Black
Alaska Native-Caucasian
Alaska Native-Unknown
Asian
Asian-Black
Asian-Caucasian
Asian-Other
Asian-Pacific Islander
Asian-Unknown
Black
Black-Caucasian
Black-Other
Black-Pacific Islander
Black-Unknown
Caucasian
Caucasian-Other
Caucasian-Unknown
Missing
Native American
Other
Other-Unknown
Pacific Islander
Pacific Islander-Caucasian
Unknown

© 2025 Nickel KB et al. *JAMA Network Open*.

eTable 2. Characteristics of healthcare-associated infections (HAIs)

Type of HAI	N	Number of days hospitalized, median (interquartile range)	Number of days hospitalized before HAI, median (interquartile range)
HAI	6,699	19 (11-31)	7 (4-13)
Blood HAI	1,572	25 (17-40)	10 (5-16)
Respiratory HAI	2,497	23 (14-38)	7 (4-13)
Urine HAI	3,146	16 (9-27)	7 (4-13)

© 2025 Nickel KB et al. *JAMA Network Open*.

eTable 3. Social determinants of health and healthcare-associated infections (HAIs)

Variable	HAI admission		Blood HAI admission		Respiratory HAI admission		Urine HAI admission	
	Yes, n (%)	No, n (%)	Yes, n (%)	No, n (%)	Yes, n (%)	No, n (%)	Yes, n (%)	No, n (%)
N	6,699	208,256	1,572	213,383	2,497	212,458	3,146	211,809
Race and Rurality								
Black, urban	1,955 (29.2)	69,436 (33.3)	430 (27.4)	70,961 (33.3)	808 (32.4)	70,583 (33.2)	903 (28.7)	70,488 (33.3)
Black, rural	45 (0.7)	1,054 (0.5)	13 (0.8)	1,086 (0.5)	23 (0.9)	1,076 (0.5)	13 (0.4)	1,086 (0.5)
White, urban	3,368 (50.3)	104,905 (50.4)	807 (51.3)	107,466 (50.4)	1,151 (46.1)	107,122 (50.4)	1,626 (51.7)	106,647 (50.4)
White, rural	1,331 (19.9)	32,861 (15.8)	322 (20.5)	33,870 (15.9)	515 (20.6)	33,677 (15.9)	604 (19.2)	33,588 (15.9)
Medicaid	1,933 (28.9)	56,898 (27.3)	466 (29.6)	58,365 (27.4)	831 (33.3)	58,000 (27.3)	802 (25.5)	58,029 (27.4)
Census median household income: Lowest national income quartile	2,567 (38.3)	78,964 (37.9)	590 (37.5)	80,941 (37.9)	1,045 (41.9)	80,486 (37.9)	1,153 (36.6)	80,378 (37.9)

© 2025 Nickel KB et al. *JAMA Network Open*.

eTable 4. Social determinants of health and adverse outcomes among healthcare-associated infection admissions

Variable	Intensive care unit admission[a]		Death	
	Yes, n (%)	No, n (%)	Yes, n (%)	No, n (%)
N	653	2,534	1,170	5,529
Race and Rurality				
Black, urban	171 (26.2)	709 (28.0)	337 (28.8)	1,618 (29.3)
Black, rural	7 (1.1)	7 (0.3)	17 (1.5)	28 (0.5)
White, urban	343 (52.5)	1,375 (54.3)	585 (50.0)	2,783 (50.3)
White, rural	132 (20.2)	443 (17.5)	231 (19.7)	1,100 (19.9)
Medicaid	179 (27.4)	673 (26.6)	291 (24.9)	1,642 (29.7)
Census median household income: Lowest national income quartile	246 (37.7)	906 (35.8)	442 (37.8)	2,125 (38.4)

[a] 3,512 healthcare-associated infection admissions were admitted to the intensive care unit (ICU) before the HAI date and were excluded from the post-HAI ICU comparison.

© 2025 Nickel KB et al. *JAMA Network Open*.
