# Supplementary material for: Intersection of Race and Rurality With Health Care–Associated Infections and Subsequent Outcomes
Source: JAMA Netw Open. 2025 Feb 3;8(2):e2453993. doi: 10.1001/jamanetworkopen.2024.53993 (PMC11791699; doi:10.1001/jamanetworkopen.2024.53993)
Supplement: Supplement 2. — Data Sharing Statement [file jamanetwopen-e2453993-s002.pdf]

Data Sharing Statement

Nickel. Intersection of Race and Rurality With Health Care–Associated Infections and Subsequent Outcomes. *JAMA Netw Open*. Published January 14, 2025. doi:10.1001/jamanetworkopen.2024.53993

Data
Data available: No
